# Five-year follow-up after a single US-guided high intensity focused ultrasound treatment of breast fibroadenoma

**DOI:** 10.1038/s41598-024-68827-4

**Published:** 2024-08-07

**Authors:** B. Boeer, E. Oberlechner, R. Rottscholl, I. Gruber, S. Guergan, S. Brucker, M. Hahn

**Affiliations:** 1 Department of Women’s Health, University Hospital of Tuebingen, Calwerstraße 7, 72076 Tübingen, Germany; 2 Department of Pathology, University Hospital of Tuebingen, Tübingen, Germany

**Keywords:** Breast fibroadenoma, US-guided high-intensity focused ultrasound (US-HIFU), Thermal ablation, Breast cancer, Clinical trials

## Abstract

The aim of this study was to evaluate the long-term efficacy of a single ultrasound-guided high-intensity focused ultrasound (US-HIFU) treatment in patients with breast fibroadenoma (FA) in terms of volume and pain reduction as well as palpation findings. From december 2013 until november 2014 27 women with a symptomatic FA were treated in one HIFU-session. Follow-up visits were performed after 7 days, 6 months and 1, 2, 3 and 5 years with clinical examination and ultrasound. One year after the procedure, a core needle biopsy of the residual lesion was offered. There was a significant volume reduction 6 months after HIFU from 1083.10 to 347.13 mm^3^ (p < 0.0001) with a mean volume reduction ratio (VRR) of 61.63%. Thereafter the FAs showed a further, but no longer significant decrease in size. One patient with an initial incomplete ablation and histologically confirmed persistent vital cells after 1 year showed a strong regrowth after 3 years. Excluding this patient from analysis, the mean VRR at months 12, 24, 36, and 60 was 86.44%, 94.44%, 94.90%, and 97.85%, respectively. Before HIFU, 59.26% of the patients had pain (22.33/100 VAS) which decreased to 6.56/100 after 12 months and remained reduced over the 5 year follow up period. A decrease in palpability from 85.19 to 7.69% was observed within 24 months. A single HIFU intervention let to a substantial reduction in size, pain, and palpability with its most potential effect during the first 12 months. Subsequently, the observed effect remained stable over a 5 year follow up period. Incomplete initial treatment was associated with the risk of regrowth.

## Introduction

Fibroadenomas (FAs) of the breast are the most common benign breast findings with an incidence of 25% and occur mainly between the age of 15 and 35^1^. Most of the time, those affected present with a (progressive) palpable finding, less often with pain or cosmetic restrictions^2,3^. Without treatment, FAs sometimes decrease in size or disappear completely, but more than half remain constant in size, and some grow significantly^2,4^. Literature on recurrence rates and the development of additional fibroadenomas is limited. However, it has been reported that FAs tend to increase in size to approximately 2–3 cm within a 12-month period and then demonstrate little further growth^5^.

Since FAs are benign, active surveillance is generally recommended in patients under 35 years of age^4^—but there exist no guidelines.

Clinical intervention is recommended for symptomatic and distressed patients or in case of an increase in size^6,7^.

Surgical excision is unequivocally indicated for enlarging lesions exhibiting associated atypia or presenting clinical, radiologic, or pathologic features raising concern for a phyllodes tumor^8,9^, despite low malignancy rates^10^.

High-intensity focused ultrasound (HIFU) is the only non-invasive alternative that can be performed on an outpatient basis under local anesthesia. It is the only non-invasive method that doesn’t need any needle or probe insertion inside the lesion and therefore leaves no scars on the skin. This thermoablative technique focuses energy on a defined area and heats it to > 65 °C, resulting in protein denaturation and coagulation necrosis^11,12^. Several clinical studies in benign and malignant tumors have shown the feasibility and safety of HIFU treatment^13–17^. So far there are only few data on the treatment of FAs of the breast^14,15,18–23^ and only one study with a long-term follow-up of 2 years^16^.

The aim of the present study was to evaluate the long-term efficacy of a single HIFU treatment with the Echopulse device (Theraclion, Malakoff, France) for symptomatic FAs in terms of volume and pain reduction as well as palpation findings over a period of 5 years. In addition, changes in subsequent mammograms due to HIFU treatment were evaluated.

## Materials and methods

### Study design

This prospective, monocentric, nonrandomized, open-label trial was conducted from december 2013 until november 2014 in Tuebingen, Germany. The study was approved by the local Ethics Committee of the University of Tuebingen (441/2013MPG23) and registered under clinicaltrials.gov NCT02011919 (registration date 1st of July 2015).

**Figure 1:**
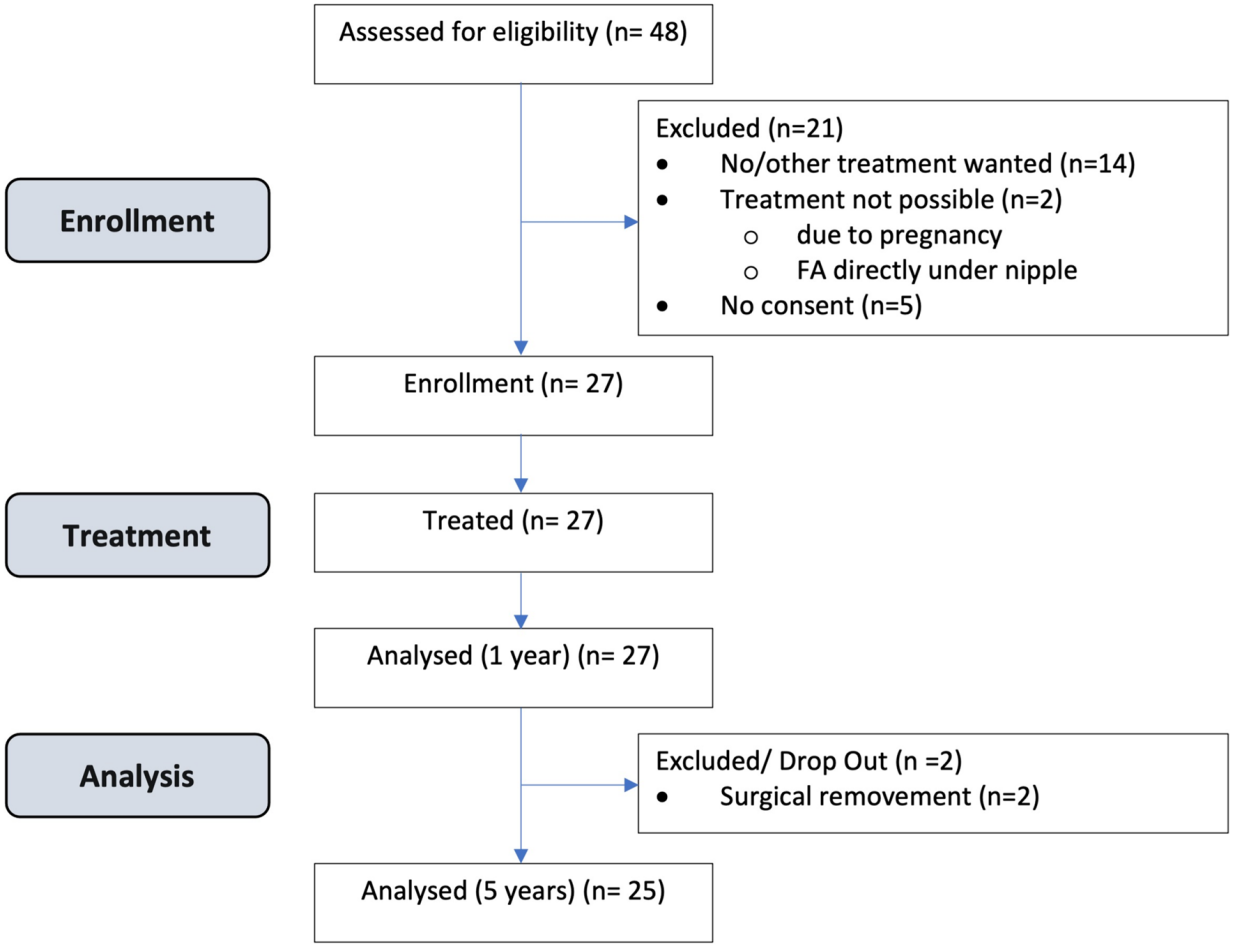
Consort diagram of patient enrollment.

48 patients with histologically confirmed FA were screened. Twenty-seven of these women (56%) fit the criteria and were enrolled in the trial (Fig. 1). 19 patients had multiple FA. In these cases, the most symptomatic FA was treated.

Follow-up visits were performed after 7 days (D1), 6 months (M6) and 1 (M12), 2 (M24), 3 (M36) and 5 (M60) years. There were two dropouts after 12 and 24 months. For the remaining 25 participants, data is available for 5 years (Fig. 1).

### Inclusion and exclusion criteria

Women aged > 18 years with a symptomatic FA histologically confirmed by core needle biopsy, with a maximum diameter of 25 mm and signed written informed consent were included in the study.

Exclusion criteria included women who were currently pregnant or breastfeeding, had received cryoablation or laser therapy on the ipsilateral side within the preceding 12 months, had breast implants or a history of breast cancer and/or radiotherapy within the previous 5 years.

### HIFU-treatment and follow-up visits

All patients received a single HIFU application under local anesthesia using the ultrasound-guided Echopulse device (Theraclion, Malakoff, France). Details to the procedure were described previously^14^ (Table S1).

**Figure 2:**
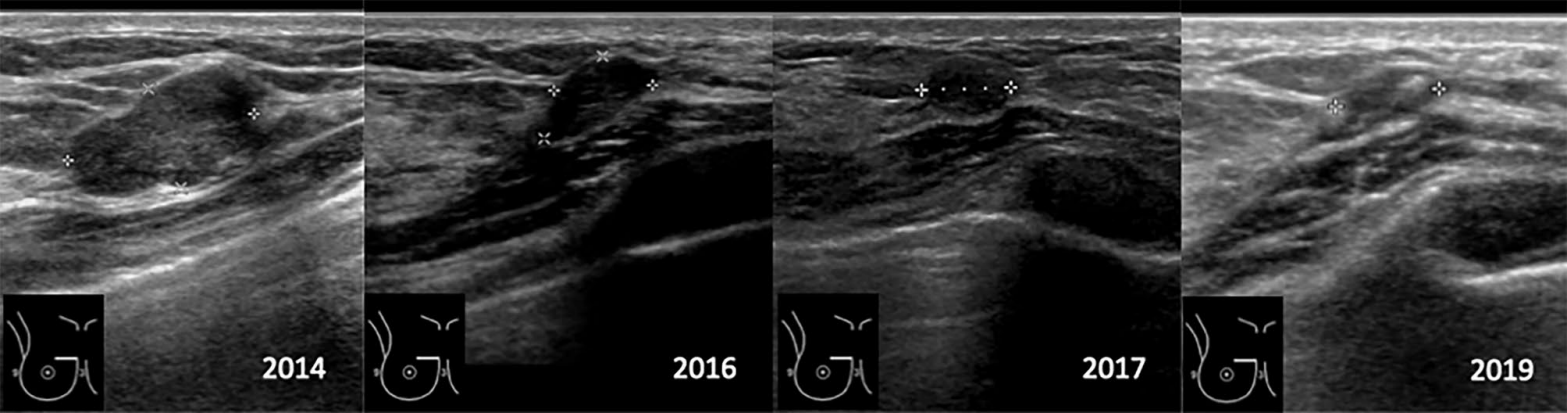
US-follow up after HIFU treatment.

Patients underwent six follow-up assessments that consisted of a physical examination, including palpation of the lump, followed by an ultrasound measurement of the lesion (Fig. 2), with volume calculation performed using the ellipsoid model (d1·d2·d3·π/6). The volume reduction ratio (VRR) was calculated by the following formula: VRR (%) = [(initial volume – final volume) × 100]/initial volume^24^.

After the age of 40, all patients were offered a mammography, which included standard mediolateral oblique (MLO) and craniocaudal (CC) views and categorized breast density. The presence of a palpable lesion was documented.

The pain level was documented using the standard Visual Analog Scale (VAS 0–100) at each visit. After 12 months, patients with sonographically indistinct residuals were offered a core needle biopsy.

All methods were performed in accordance with the relevant guidelines and regulations.

### Data collection and statistical analysis

All data was documented in case report forms and captured in a database; therefore, double data entry was performed.

Descriptive data are presented as mean, 95% confidence interval and standard deviation or median, range and interquartile range, as appropriate according to the distribution. Categorical variables are compared using the chi-square test or Fisher’s exact test. Continuous data with normal distribution were analyzed by an independent sample T-test; and data with skew distribution were analyzed by the Wilcoxon rank-sum test. Comparisons of multiple variables were performed using ANOVA test. Correlations were calculated with the Pearson correlation test. Data were analyzed using the JMP-software version 16.1.0 (SAS Institute Inc.) and Prism for macOS version 10.1.0 (GraphPad software LLC). The significance was set at 0.05.

## Results

### Patient characteristics

27 women (mean age 28.85 [SD 9.06], mean BMI of 22.24 kg/m^2^ [SD 2.35]), with one symptomatic FA were included in the study. In case of multiple lesions only one histologically confirmed and symptomatic FA received a single US-guided HIFU treatment under local anesthesia (Table 1; Fig. 1).

**Table 1:** Basic characteristics of patients (n = 27) and of breast fibroadenomas (n = 27).

Patient characteristics^a^	N (%)	mean (± SD)	Min–max
Age (years)^a^	27	28.85 (9.06)	18.00–50.00
BMI (kg/m^2^)^a^	27	22.24 (2.35)	17.67–29.06
Breast density^a^ (mammography) (A–D)	27		
Scattered density (B)	3 (11.11%)		
Heterogeneous density (C)	4 (14.81%)		
Extremely dense (D)	20 (74.07%)		
Cup size (A–E)^a^	27		
Cup A	1 (3.70%)		
Cup B	13 (48.15%)		
Cup C	10 (37.04%)		
Cup D	1 (3.70%)		
Cup E	2 (7.41%)		
Pain (D0 pre-HIFU)^a^	27		
Pain	16 (59.26%)		
No pain	11 (40.74%)		
PAIN VAS 0–100	27	22.33 (23.47)	0.00–71.00
Tumor characteristics^b^			
Fibroadenoma (D0 pre-HIFU)^b^	27		
Palpable finding	23 (85.19%)		
Not palpable	4 (14.81%)		
Fibroadenoma (D0 pre-HIFU)^b^	27		
Volume (mm^3^)	27	1083.10 (801.02)	108.07–3138.12
Longest diameter (mm)	27	14.57 (4.30)	6.40–24.90
Interventions^c^	23		
CNB after 1 year	21 (91.30%)		
Excision in follow-up	2 (8.69%)		

^a^Data on patient characteristics are presented in terms of number (n), percentage (%), mean (± SD), minimum (Min), and maximum (Max).

^b^Data on tumor characteristics are presented in terms of number (n), percentage (%), mean (± SD), minimum (Min), and maximum (Max).

^c^Data on interventions are presented in terms of number (n) and percentage (%).

The mean duration of the procedure was 38 min. 21 persons had a core needle biopsy done after 1 year. Vital cells were still found in three probes.

The mean longest diameter of the untreated FAs was 14.57 mm [SD 4.30], assessed by ultrasound, with a range of 6.4 to 24.9 mm. The mean volume of the FAs before HIFU treatment (D0) was 1083.07 mm^3^ (Tables 1 and 2).

**Table 2:** Volume reduction of fibroadenomas after HIFU-intervention.

Visit	FU^a^	Pts/FA^b^	Volume^c^ (mm^3^)	VRR^d^ (%)	VRR^e^ (%)
V1	D0	27/27	1083.10 ± 801.02	–	–
V2	D7	27/27	1244.53 ± 1317.63	− 9.64% ± 70.60%	− 9.67% ± 72.00%
V3	M6	27/27	347.13 ± 389.22	61.63% ± 47.21%	62.30% ± 48.01%
V4	M12	27/27	203.15 ± 316.21	84.81% ± 17.35%	86.44% ± 15.45%
V5	M24	26/26	117.16 ± 260.44	92.04% ± 15.66%	94.44% ± 9.95%
V6	M36	21/21	124.29 ± 282.00	92.05% ± 16.35%	94.90% ± 10.07%
V7	M60	25/25	129.65 ± 465.27	92.17% ± 29.09%	97.85% ± 6.41%

The data represent the mean values ± SD for the variables *Volume* and *VRR* (Volume Reduction Rate).

^a^The follow-up (FU) was conducted during visits (V1-V7) on Day 0 (D0), Day 7 (D7) post-HIFU, and at 6, 12, 24, 36, and 60 months (M6, M12, M24, M36, M60) after the HIFU intervention.

^b^Number (n) of examined patients (Pts) and fibroadenomas (FA) at each visit (V1-V7).

^c^Volume (mean ± SD) of fibroadenomas before HIFU (V1) and after HIFU at visits V2-V7.

^d^Volume Reduction Rate (VRR) in % (mean ± SD) of fibroadenomas after HIFU at visits V2-V7.

^e^Volume Reduction Rate (VRR) in % (mean ± SD) of fibroadenomas after HIFU at visits V2-V7 after excluding patient No.22.

### Follow-up characteristics

Follow-up visits were scheduled at 6 months and then at annual intervals after the HIFU intervention (Table 2). After two patients had their FA surgically removed after 1 and 2 years respectively, data was only available from 25 patients in the subsequent follow-up (M36-M60).

**Figure 3:**
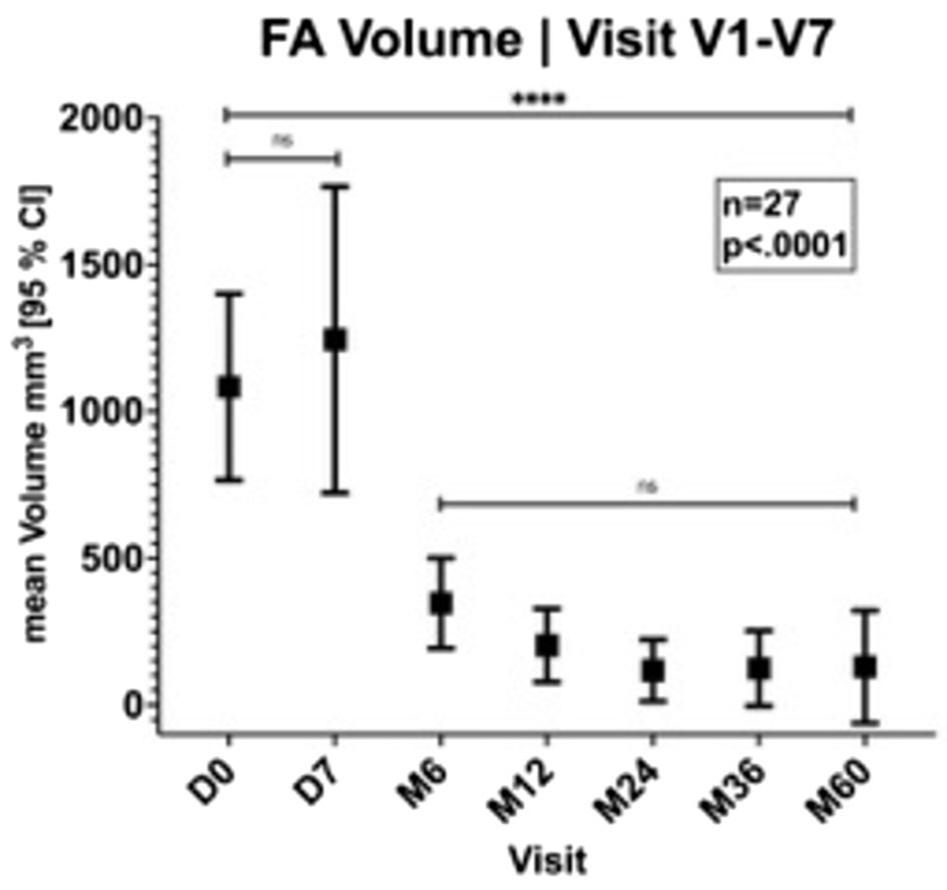
Volume reduction after HIFU intervention.

After an initial increase in swelling 7 days after HIFU treatment, a significant reduction in volume to a mean volume of 347.13 mm^3^ was observed 6 months (M6) post-intervention, resulting in a mean VRR of 61.63%. In the subsequent follow-up, 6 to 60 months after the HIFU, a further, albeit non-significant, volume reduction of the FAs was observed. After 5 years (M60) the mean volume was 129.65 mm^3^ (Table 2; Fig. 3).

The mean VRR after 12, 24, 36 and 60 months post-HIFU was 84.81%, 92.04%, 92.05% and 92.17% respectively (Table 2).

**Figure 4:**
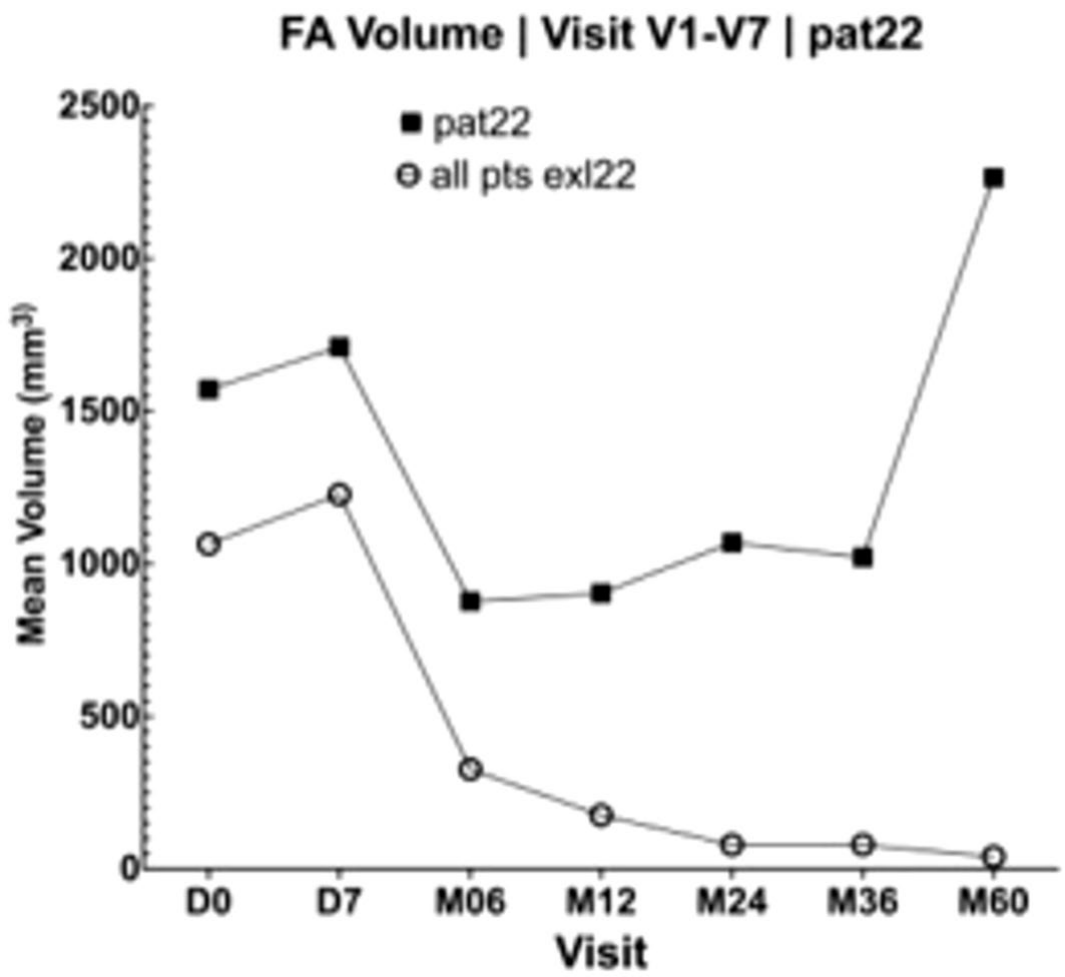
FA volume in patient no. 22 (n = 1) compared to the other patients (n = 26).

One patient with suspected inadequate therapeutic response of a lobulated fibroadenoma (no. 22; Fig. 4), with histologically confirmed viable cells in core needle biopsy after 1 year, had a substantial volume reduction within the first 3 years post-HIFU from 1570.57 mm^3^ (D0) to 1021.11 mm^3^ (M36). Subsequently, a substantial increase in size 2264.11 mm^3^ was observed at the 5-year follow up (M60). Excluding this patient from the analysis, the mean VRR would be 94.90% after 3 years and 97.85% at the 5-year follow-up (Table 2).

### Pathological findings

Two patients (no. 3 and 21) had core needle biopsy 1 year after HIFU-treatment and underwent surgical resection in the follow up due to pain in the treated area.

**Figure 5:**
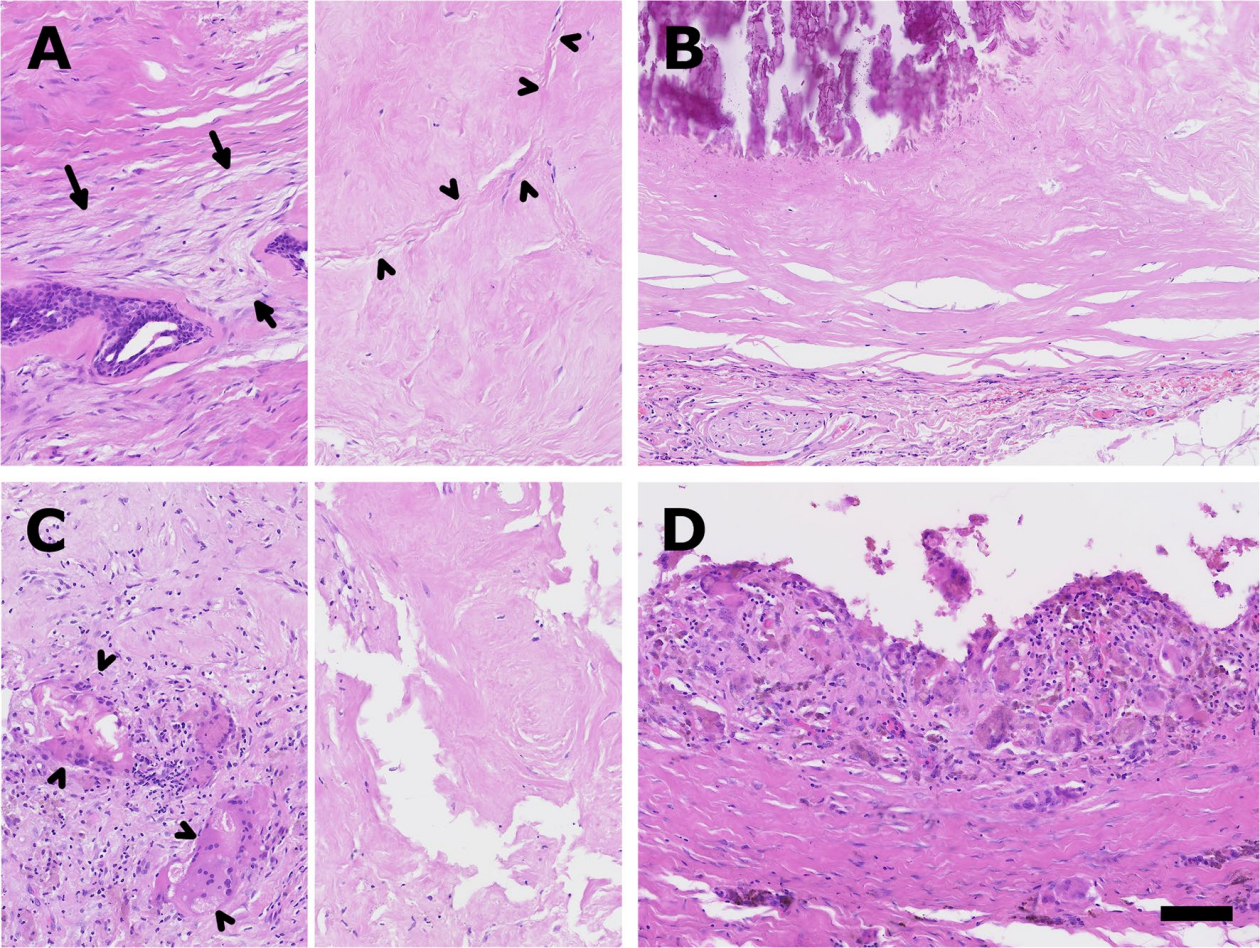
Histopathological findings. Representative photomicrographs show core needle biopsies (**A**,**C**) and the corresponding resection specimen (**B**,**D**) of patient no. 21 (upper part) and patient no. 3 (lower row) after HIFU treatment.

Patient no. 21 had a core needle biopsy done for a residual lesion of 10 × 7 × 10 mm (Fig. 5A). It mainly showed two patterns; first: loose fibrosis with fibroblastic proliferation (arrow, left half) and two single remaining ducts of the fibroadenoma and second: a dense nearly acellular fibrosis with fibrosis of the former epithelium containing clefts of the regressive parts of the fibroadenoma (arrowhead, right half). The corresponding resection specimen (Fig. 5B) 6 months later showed sharply demarcated fibrosis with opaque calcifications (upper left part of the image) and no remaining epithelium of the fibroadenoma.

The histopathological specimen of patient no. 3 (Fig. 5C and D) with a residual lesion measuring 7 × 6 × 9 mm 1 year after treatment showed giant cell reaction (arrowhead, left half) and dense fibroses (right half) on the core needle biopsy (C) and a pseudocystic lesion with organizing granulation tissue and giant cell reaction in the resection specimen (D) 11 months later (scale bar 100um, 20x).

### Pain level

Initially, 59.26% of the women experienced pain in the area of the FA (16/27). The mean pain level given by the 16 participants before the intervention was 22.33 (SD 23.41) on a 100-part visual analogue scale (VAS). One year after the HIFU intervention, the average pain score was 6.56 (SD 3.41), indicating a significant decrease in pre-interventional pain symptoms (p < 0.0001).

**Figure 6:**
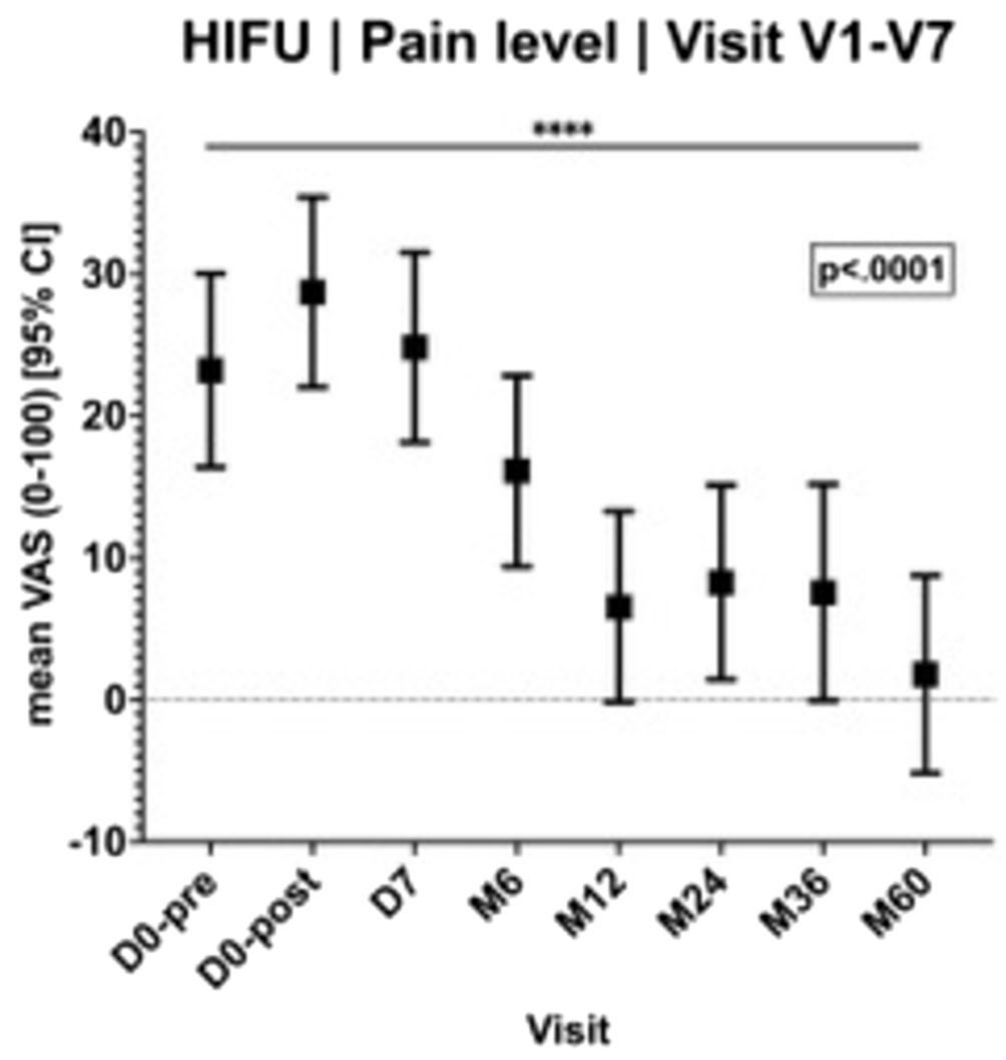
Pain in the follow-up after HIFU.

At the follow-up examinations 12 to 60 months after the intervention, the pain symptoms reported by the patients with a score of 8.27 (SD 3.41) after 2 years were up to 1.80 (SD 3.54) at the end of the study in a constantly low and not significantly different level (Fig. 6).

### Palpable and cosmetic findings

Prior to HIFU treatment, 23 out of 27 patients exhibited palpable findings (85.19%). At 12 months, nine lesions remained palpable, followed by two at 24 months (7.69%), and one of 21 (4.76%) at 36 months. After HIFU intervention, a marked alteration in palpable findings was observed within the initial 2 years, decreasing from 85.19 to 7.69% within 24 months (Fig. S1). There were no delayed reactions to HIFU in the form of hyperpigmentation.

### Mammography after HIFU

Eight patients over the age of 40 underwent mammography after HIFU treatment as part of their follow-up. Of these, one was assessed as BIRADS 1 (no negative finding on breast imaging reporting and data system), 75% were assessed as BIRADS 2 (a benign category in breast imaging reporting and data system) and one as BIRADS 3 (probably benign), focused on the treated fibroadenoma area.

## Discussion

The current evaluation, undertaken 5 years following a single US-controlled HIFU treatment of symptomatic FAs, reveals that a substantial reduction in volume is achieved within the initial 6 months post-intervention (mean VRR 68%). Subsequently, within the following years there is a further, but no longer significant volume reduction with a constant low target volume. A HIFU treatment thus represents a long-term effective treatment for symptomatic FA with a mean volume reduction between 92.2 and 97.9% over 5 years.

This volume reduction may be even greater in a larger cohort, as there seems to exist a learning curve with a significant improvement of the mean Volume Reduction Ratio (VVR) and treatment time after 40–60 HIFU sessions^25,26^.

Our results for the first year (mean VRR of 84.3%) are consistent with the literature (Kovatcheva, 72.5% after 12 months^15^; Kwong, 62% after 12 months^23,27^). In the study with the longest follow-up to date, Kovatcheva et al.^16^ were able to achieve a volume reduction of 77.3% after a single US-controlled HIFU treatment in 19 women 24 months after HIFU treatment. However, if a second HIFU treatment was carried out 6–9 months later due to insufficient volume reduction (i.e. < 50% or a remaining volume > 1.5 ml), there was a mean volume reduction of 90.5% after 24 months (n = 7). In both groups, the FA volume decreased significantly at the 1-month (p < 0.001) and 3-month follow-up (p = 0.005) and continued to decrease up to the 24-month endpoint (p < 0.001 and p = 0.003). Kovatcheva et al. postulated that due to the size reduction, a further volume reduction should also be expected after 24 months.

Nevertheless, as seen in case no. 22 in this study, an incomplete treatment leads to initial cell shrinkage due to denaturation and necrosis of the treated area. However, viable cells can still persist and have the potential to grow.

As Peek et al.^21^ performed only circumferential ablation of the FAs to reduce treatment times, long-term results would be very interesting as they initially reported a volume reduction of 43.2% (SD 35.4%; p < 0.005, paired t-test) on US at 12 months post-treatment.

There is currently no available information on the length of time required for the body to fully absorb necrotic material or the initial size limitation before complete absorption is no longer possible. Kwong et al. defined treatment failure as no tumor shrinkage or increase in tumor volume at 12 months post HIFU treatment^27^ which occurred in 4 of their 60 cases (6.7%).

Since the treated tissue is not excised after treatment, the volume calculation is based on sonographic measurements in the further course.

In the present study, the treatment itself and the subsequent follow-up controls were carried out by ultrasound, primarily by one examiner to prevent interobserver variability. Nevertheless, it is often difficult to differentiate between necrotized or scarred avital fibroadenoma and regular breast parenchyma or lipoid necrosis.

Therefore, the question is to which extent the treated tissue is completely resorbed or, rather, is the scar integrated into the surrounding tissue or remodeled or replaced by fibroblastic tissue. Liang et al. accessed the nonperfused volume of treated FAs by contrast-enhanced ultrasound or contrast-enhanced MRI after 3 months^24^.

In the present study a core needle biopsy was done in most patients 1 year after the HIFU-ablation and revealed three treated FAs with vital FA-cells (no. 15, 21 and 22).

Although vital fibroadenoma cells were still detected in patient no. 15 1 year after ablation, the treated lesion resolved completely on US in the further follow-up.

In patient no. 21 single remaining ducts of the fibroadenoma were detectable in the biopsy. At the time of the surgical resection 6 months later the lesion was completely sclerotic and no vital fibroadenoma was detectable, but the patient still had pain.

Case no. 22 showed that incomplete initial ablation with persistent vital fibroadenoma cells followed by initial reduction can lead to long-term regrowth.

A consistent histopathologic feature of the two cases which subsequently underwent surgical removal (no. 3 and 21) was dense fibrosis in the core needle biopsy. Case no. 3 showed a pseudocystic mass with organizing reaction in the wall of the lesion. This inflammation was already present in the core needle specimen, but it was not a dominant feature.

Thus, in case no. 21 the complete sclerosis/scaring of the lesion seemed to be the endpoint, while in the other case the remodeling of the tissue was still going on at the time of the resection. Thus, making it likely that the lesion would have shrunk further.

These cases impressively illustrate different imaging, histopathological and clinical outcomes following one single HIFU treatment.

In general, the extent to which volume reduction should be used as the main surrogate needs to be reconsidered, as the clinical benefit of treating benign FAs should rather be assessed in terms of pain, cosmetic outcome and quality of life^23^.

In the current study, a substantial reduction in pain was observed as early as 12 months following the HIFU intervention (score 6.6) and remained consistently low throughout the 5-year study period.

The initially concerning palpable findings were significantly less frequent at the 24-month mark following treatment; however, thereafter, no further significant changes were observed.

Within the 5-year period, there were no delayed cosmetic reactions to HIFU. The mammographic findings after HIFU were comparable to those after surgery.

## Conclusion

Compared to the gold standard of surgical excision and in contrast to US-guided vacuum-assisted biopsy or to other ablative procedures, HIFU is currently the only completely non-invasive treatment method and therefore rapidly growing in popularity. It represents a good alternative in the treatment of FA as it can be performed safely under local anesthesia and leads to a reduction in symptoms and volume even over a 5 year follow up.

As incomplete initial HIFU-treatment is associated with the risk of regrowth, annual sonographic follow-up is recommended for a minimum of 3 years.

### Clinical relevance statement

To date, there are no long-term data on the effectiveness of high intensity focused ultrasound treatment of fibroadenomas. After insufficient treatment with remaining vital cells, in selected cases fibroadenomas can continue to grow. Annual US monitoring for at least 3 years is recommended.

### Supplementary Information

Supplementary Figure S1.

Supplementary Table S1.

## Data Availability

The datasets used and analyzed during the current study are available from the corresponding author on reasonable request.
