# Supplementary figures and images for: Five-year follow-up after a single US-guided high intensity focused ultrasound treatment of breast fibroadenoma
Source: Sci Rep. 2024 Aug 7;14:18370. doi: 10.1038/s41598-024-68827-4 (PMC11306253; doi:10.1038/s41598-024-68827-4)

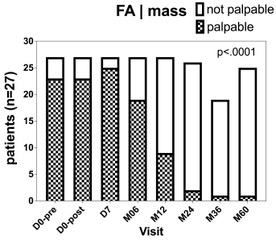


Figure S1. Change in the palpable findings

Supplement: Supplementary file 1 — Supplementary Figure S1. [file 41598_2024_68827_MOESM1_ESM.docx]
