# Supplementary material for: Five-year follow-up after a single US-guided high intensity focused ultrasound treatment of breast fibroadenoma
Source: Sci Rep. 2024 Aug 7;14:18370. doi: 10.1038/s41598-024-68827-4 (PMC11306253; doi:10.1038/s41598-024-68827-4)
Supplement: Supplementary file 2 — Supplementary Table S1. [file 41598_2024_68827_MOESM2_ESM.docx]

**Table S1: The thermoablative HIFU-procedure**

*Hahn M, Fugunt R, Schoenfisch B, Oberlechner E, Gruber IV, Hoopmann U, et al.*

*High intensity focused ultrasound (HIFU) for the treatment of symptomatic breast fibroadenoma. Int J Hyperthermia. 2018;35(1):463-70.*

Depending on the fibroadenoma's location, patients were positioned either supine or laterally, with a breast compression paddle in place to prevent uncontrolled movement of the fibroadenoma. With a high-resolution handheld probe, the fibroadenoma was located and delineated on the US screen while the software calculated the overall energy per lesion and the number of pulses.

The thermoablative HIFU-procedure started with a single pulse of 40 watts (peak intensity at focus 27 kWatts/cm2) and could be reduced stepwise to a minimum of 30 watts (peak intensity at focus 20 kWatts/cm2), if necessary, for pain control. The energy was applied stepwise using numerous pulses. The treatment head moved automatically until the whole FA volume was covered. Treatment started with consecutive repeated HIFU pulses and cooling pauses. The derated power for treatment at a certain depth was calculated by the system automatically and it applied the acoustic power to the HIFU transducer. The mean energy for the complete HIFU treatment was 6.2 kiloJoules (SD 2.7, median 6.5 min 1.2, maximum 11.3 kiloJoules). The mean maximum power during treatment was 41 Watts (SD 3, median 40, minimum 40, maximum 50 Watts).
